# Perfusion-Weighted Imaging: The Use of a Novel Perfusion Scoring Criteria to Improve the Assessment of Brain Tumor Recurrence versus Treatment Effects

**DOI:** 10.3390/tomography9030087

**Published:** 2023-05-23

**Authors:** Sneha Sai Mannam, Chibueze D. Nwagwu, Christina Sumner, Brent D. Weinberg, Kimberly B. Hoang

**Affiliations:** 1 Perelman School of Medicine, University of Pennsylvania, Philadelphia, PA 19104, USA; 2 Department of Neurosurgery, School of Medicine, Emory University, Atlanta, GA 30322, USA; chibueze.dominic.nwagwu@emory.edu (C.D.N.); kimberly.bojanowski.hoang@emory.edu (K.B.H.); 3 Department of Radiology and Imaging Sciences, School of Medicine, Emory University, Atlanta, GA 30322, USA; christina.schmidt.sumner@emory.edu (C.S.); brent.d.weinberg@emory.edu (B.D.W.)

**Keywords:** perfusion-weighted imaging, malignant brain tumor recurrence, radiation treatment effects, neuroradiology, perfusion scoring criteria

## Abstract

Introduction: Imaging surveillance of contrast-enhancing lesions after the treatment of malignant brain tumors with radiation is plagued by an inability to reliably distinguish between tumor recurrence and treatment effects. Magnetic resonance perfusion-weighted imaging (PWI)—among other advanced brain tumor imaging modalities—is a useful adjunctive tool for distinguishing between these two entities but can be clinically unreliable, leading to the need for tissue sampling to confirm diagnosis. This may be partially because clinical PWI interpretation is non-standardized and no grading criteria are used for assessment, leading to interpretation discrepancies. This variance in the interpretation of PWI and its subsequent effect on the predictive value has not been studied. Our objective is to propose structured perfusion scoring criteria and determine their effect on the clinical value of PWI. Methods: Patients treated at a single institution between 2012 and 2022 who had prior irradiated malignant brain tumors and subsequent progression of contrast-enhancing lesions determined by PWI were retrospectively studied from CTORE (CNS Tumor Outcomes Registry at Emory). PWI was given two separate qualitative scores (high, intermediate, or low perfusion). The first (control) was assigned by a neuroradiologist in the radiology report in the course of interpretation with no additional instruction. The second (experimental) was assigned by a neuroradiologist with additional experience in brain tumor interpretation using a novel perfusion scoring rubric. The perfusion assessments were divided into three categories, each directly corresponding to the pathology-reported classification of residual tumor content. The interpretation accuracy in predicting the true tumor percentage, our primary outcome, was assessed through Chi-squared analysis, and inter-rater reliability was assessed using Cohen’s Kappa. Results: Our 55-patient cohort had a mean age of 53.5 ± 12.2 years. The percentage agreement between the two scores was 57.4% (κ: 0.271). Upon conducting the Chi-squared analysis, we found an association with the experimental group reads (*p*-value: 0.014) but no association with the control group reads (*p*-value: 0.734) in predicting tumor recurrence versus treatment effects. Conclusions: With our study, we showed that having an objective perfusion scoring rubric aids in improved PWI interpretation. Although PWI is a powerful tool for CNS lesion diagnosis, methodological radiology evaluation greatly improves the accurate assessment and characterization of tumor recurrence versus treatment effects by all neuroradiologists. Further work should focus on standardizing and validating scoring rubrics for PWI evaluation in tumor patients to improve diagnostic accuracy.

## 1. Introduction

Magnetic resonance imaging (MRI) remains the conventional non-invasive modality in assessing the disease progression and treatment response of central nervous system (CNS) tumors [1]. Emerging advanced MRI modalities or advanced brain tumor imaging modalities such as diffusion-weighted imaging (DWI), spectroscopy, and perfusion-weighted imaging (PWI) are often incorporated into the clinical setting to assist in challenging diagnoses, especially given their unique abilities to characterize tumoral and peritumoral tissue microstructure and metabolite composition [2,3,4]. In particular, PWI has been utilized as a tool to further narrow the differential diagnosis of primary CNS lesions and metastases, guide further diagnostic and treatment approaches, and monitor disease progression [5]. Specifically, there is utility in the post-treatment setting in discerning radiation necrosis and pseudo-progression from disease progression or treatment failure when combined with other MRI sequences. For example, studies in the literature demonstrate that by utilizing relative cerebral blood volume (rCBV) values from PWI scans, it is possible to distinguish enhancing neoplastic lesions from enhancing lesions of non-neoplastic origin. For example, the literature findings show that in using relative cerebral blood volume (rCBV) values of PWI scans, enhancing neoplastic lesions could be differentiated from non-neoplastic enhancements [6,7]. 

One of the great diagnostic challenges in longitudinal brain tumor management is the differentiation of tumor progression versus treatment effect, particularly in the post-radiation setting. Despite the utilization of many modalities including DWI to distinguish between these two entities, considerable clinical difficulties remain with non-invasive imaging alone. Invasive modalities such as biopsy and/or resection of these lesions for pathologic confirmation remain the only definitive methods of diagnosis in many cases. This inability to reliably identify disease progression affects all aspects of long-term management, resulting in increased imaging frequency and number, delayed initiation of salvage therapy in the setting of recurrence, increased duration of neurological symptoms in certain patients, and interventions such as biopsy and surgical resection to make a correct diagnosis to guide further therapy. 

The complexity of the underlying pathophysiology as well as variability in how PWI is interpreted can contribute to inconsistencies in PWI results, which in turn may diminish the overall usefulness of these studies. Although PWI has been reported to be a good marker of indicating recurrence [8,9], many in clinical practice acknowledge that PWI interpretation can be unreliable and no surrogate for tissue diagnosis [10]. This variance in PWI in the neuro-oncologic setting and its subsequent effect on the predictive value has not been studied, nor have dedicated and standardized perfusion scoring criteria been created. 

Given the complexity of perfusion scan interpretations of previously irradiated brain lesions, this study evaluates the utility of a novel PWI scoring rubric created by our group as a supplemental tool for neuroradiologists to improve diagnostic accuracy. The authors aim to assess the validity of the scoring rubric by comparing the study group evaluations to matched pathologic biopsy or tissue resection results at a large tertiary care institution with significant recurrent tumor volume.

## 2. Materials and Methods

### 2.1. Study Design

This retrospective cohort study was conducted at multiple Emory University Healthcare hospitals between 2012 and 2022. The study protocol received approval from the institutional review board at Emory University, and an informed consent waiver was obtained.

We collected data from the CNS Tumor Outcomes Registry at Emory (CTORE), a prospectively managed patient outcomes database for central nervous system (CNS) tumors treated at participating sites. The study cohort consisted of patients treated at two tertiary, academic referral hospitals, as well as two mid-sized community hospitals. Our group pooled all patients within the Emory University Hospital system who exhibited suspicion of tumor recurrence in the irradiated area and required re-operation. From this patient population, we included those who had undergone pre-operative perfusion-weighted imaging (PWI) with dynamic susceptibility perfusion (DSC) for data collection and subsequent analyses. The eligibility criteria included patients aged 18 years and older with a history of previously radiated malignant brain tumor and subsequent progression of the contrast-enhancing disease, raising concerns about treatment effects versus tumor recurrence. Specifically, we focused on cases that underwent re-resection following imaging studies of the lesion. All included cases had pre-operative magnetic resonance imaging (MRI) scans, which encompassed PWI evaluation.

The cohort was stratified based on patients with a previous history of gliomas or metastasis. The primary outcome of interest was the tumor percentage of the total surgical specimen, as reported by pathology. The pathology reports classified residual tumor contents into three categories: ≤25%, 26–75%, and >75%. These categories were aligned with the corresponding perfusion reads graded by the neuro-radiologists as low, intermediate, and high, respectively.

### 2.2. Clinical Characteristics

We used electronic medical records to retrospectively collect patient information regarding age, gender, presenting symptoms, pre-operative BMI, pre- and post-operative Karnofsky performance scale (KPS) and modified Rankin scale (MRS), prior systemic therapies, prior radiation dosing and fraction number, post-surgical therapies, post-operative complications, and histopathological findings. 

### 2.3. Imaging Results and Perfusion Scoring

Each patient who underwent re-resection had two separate perfusion ratings assigned. The first perfusion rating (the control group) was assigned based on the radiology report generated at the time of pre-operative study evaluation by the neuro-radiology faculty at Emory University School of Medicine. The control group comprised neuroradiologists who subjectively assessed the perfusion levels in the radiology reports available in the electronic medical records. These reports were retrospectively retrieved and analyzed by our research team at CTORE. Based on the text in the radiology report, each study was characterized as high, intermediate, or low perfusion based on the interpretation intended by the initial prospective reader. This classification was subsequently validated by an attending neurosurgeon and a radiology resident, who independently reviewed the electronic medical record interpretations to determine the extent to which the radiologists’ assessments aligned with the progression versus pseudo-progression criteria.

**Figure 1:**
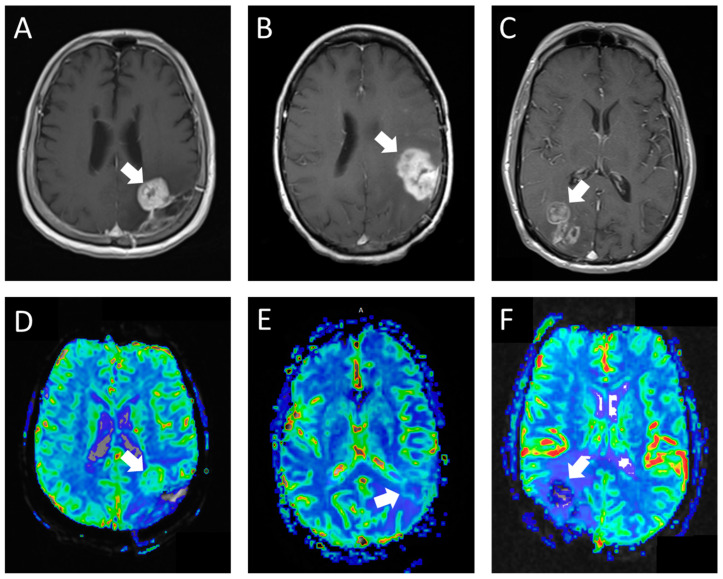
T1 post-contrast (**A**) and rCBV (**D**) in a patient with glioblastoma showing a nodule with marked enhancement and perfusion similar to areas of the normal cortex (hyperperfusion). The reresection demonstrated recurrent glioblastoma. T1 post-contrast (**B**) and rCBV (**E**) in a patient with glioblastoma showing a mass with marked solid enhancement and perfusion between the normal cortex and white matter (intermediate perfusion). The reresection demonstrated recurrent gliosarcoma with 60% tumor. T1 post-contrast (**C**) and rCBV (**F**) in a patient with glioblastoma showing heterogeneous areas of enhancement with perfusion similar to areas of normal white matter (hypoperfusion). The reresection demonstrated reactive brain tissue with organizing hematoma and the therapy effect.

The second perfusion rating (the experimental group) was assigned in a blinded retrospective review by a fellowship-trained neuroradiologist, who also played a crucial role in developing the grading rubric for the experimental group. This neuroradiologist employed our novel perfusion scoring rubric (Table 1; Figure 1) to determine the perfusion scores. The structured criteria offered benchmarks for areas of high perfusion (normal cerebral cortex) and low perfusion (normal cerebral white matter). Studies predominantly resembling the cerebral cortex in tissue characteristics were classified as high perfusion, whereas those with perfusion levels between the cortex and white matter or with only limited tumor portions exhibiting rCBV similar to the cortex were deemed intermediate perfusion. Studies where the majority of the tumor resembled white matter were categorized as low perfusion. In cases where the studies were constrained by artifacts or technical failures, they were designated as not interpretable.

To account for potential bias in the experimental group, the neuroradiologist evaluating the experimental group was blinded to the control group’s perfusion ratings. Additionally, the same cases were used for both the control and experimental groups to ensure consistency and comparability.

Intra-reader agreement was assessed by having the neuroradiologist involved in the experimental group re-read a subset of cases after a certain period to evaluate the consistency of the perfusion scores assigned using the novel perfusion scoring rubric. This additional analysis provides a more sensitive and specific measure to examine the potential effect of the rubric on PWI scoring.

### 2.4. Statistical Analysis 

Statistical analysis was performed using JMP Pro 16 (SAS Institute, Cary, NC, USA) and R 4.1.3 (R Core Team, Vienna, Austria). Descriptive statistics were performed for pre-, intra-, and post-operative characteristics of our patient cohort as well as all demographic data. For variables that had their normality assumptions met, means with standard deviations were reported; for those that did not meet the normality assumptions, medians with interquartile ranges were reported. 

A Chi-squared or Fisher’s exact test was performed to assess the accuracy of the non-specialized neuroradiology reads as well as the specialized neuroradiology reads in predicting the true percentage of tumors from the PWI. If the assumptions of the Chi-squared test of at least 80% of cells having an expected value greater than 5 and no cells having an expected value less than 1 were met, we reported the Pearson *p*-value. If these assumptions were not met, the two-sided *p*-value from the Fisher’s exact test was reported. Statistical significance was set at an alpha value of 0.05.

Inter-rater reliability between the radiologists was calculated using Cohen’s kappa.

## 3. Results

A total of 55 patients were included in our study after meeting the eligibility criteria. Our patient cohort had a mean age of 53.5 ± 12.2 years (Table 2), with 32 (58.2%) male patients. A total of 25 (45.5%) patients were asymptomatic upon imaging progression, 17 (30.9%) patients had some symptoms such as altered mental status or slight neurological deficits, and 13 (23.6%) patients had major symptoms such as seizures and major neurological deficits.

Upon PWI interpretation of the control group (Table 3), 38 (69.1%) patients were determined to show high perfusion, 10 (18.2%) had intermediate perfusion, and 6 (10.9%) had low perfusion. Upon PWI interpretation of the experimental group (Table 2), 26 (47.3%) patients were determined to show high perfusion, 18 (32.7%) had intermediate perfusion, and 11 (20%) had low perfusion. The percentage agreement between the two radiology reads was 57.4% and Cohen’s Kappa was 0.271, which indicates fair agreement.

Following PWI, 50 (90.0%) patients underwent craniotomies for resection, while 5 (9.1%) patients underwent biopsies. Of the patients who underwent craniotomies, 27 (54.0%) achieved gross-total resection (GTR). Upon histopathological analysis of the biopsied brain tissues, 18 (32.7%) samples had at most 25% tumor, 24 (43.6%) samples had between 26 and 75% tumor, and 13 (23.6%) samples had greater than 75% tumor. 

Upon analysis, we discovered a significant association (Table 4 between the pre-operative perfusion reads evaluated by the experimental group (*p*-value: 0.014) and the post-operative tumor content in the pathology report, differentiating between tumor recurrence and treatment effects. In contrast, we observed no association (Table 3) between the perfusion reads for the control group (*p*-value: 0.734) and the ability to differentiate between tumor recurrence and treatment effects. We further stratified the cohort by the previous history of metastasis or glioma (Table 2 and Table 3). We again found a significant association (Table 2) between the perfusion reads for the experimental group predicting the percentage of tumor stratified by the previous history of glioma (*n* = 42, *p*-value: 0.023). However, there was no association between the perfusion reads for the experimental group in predicting the tumor percentage stratified by their previous history of metastasis (*n* = 12, *p*-value: 0.636). In comparison, there was no association (Table 3) between the perfusion reads for the control group in predicting the tumor percentage stratified by the previous history of glioma (*p*-value: 0.661) or metastasis (*p*-value: 0.849). After stratifying with 0.5, 1, 1.5, and 2 years, we found no statistically significant evidence that the time between radiation and the perfusion scan predicted the tumor percentage. 

### Clinical Case Example

This case is presented as an example of varying PWI readings between the control and experimental groups as it relates to biopsy findings. 

A 66-year-old right-handed male with a previously resected right temporal IDH WT, unmethylated anaplastic astrocytoma, and post-proton-radiotherapy status presented with focal seizures. Repeat MRI demonstrated an increase in contrast enhancement at the primary site, initially favoring the increasing burden of the tumor. Further perfusion-weighted imaging read for the control group characterized areas of the lesion as having abnormal hyperperfusion (rCBV). Conversely, the experimental group using the grading rubric characterized areas of the lesion as being hypoperfused. Consequently, pathological findings, which were significant for treatment-related effects, coincided with the latter PWI read for the experimental group. 

## 4. Discussion

Our study demonstrated considerable variation between interpretations of perfusion-weighted imaging provided during the course of normal interpretation without additional guidance versus those provided using a specifically structured rating scale. These ratings differed in their accuracy of assessing recurrent tumor versus treatment effects using PWI, highlighting the need for methodical evaluation of perfusion imaging in the case of previously treated tumors. These results suggest that using a structured grading scale would more reliably predict tumor recurrence, thereby conserving surgical tissue sampling (and its inherent risks) for cases where recurrence is truly present (salvage therapy) or only symptomatic radiation necrosis. A standardized grading scale would also better guide systemic and re-irradiation therapies and non-invasively monitor disease progression and the long-term administration of medications such as steroids. 

After stratification of our data, our hypothesis is supported for the tissue samples of patients with a previous history of glioma but not for metastasis. Our conclusion may be limited by the small sample size of our metastasis cohort, so future studies should focus on assessing whether our grading scale is supported for patients with a history of metastases. This is obviously a cohort where the determination of treatment effect versus tumor recurrence is paramount given the increasing prevalence of brain metastases and widespread radiation treatment. 

Given the general difficulty in distinguishing treatment effects with recurrent tumors in the immediate post-treatment period (3 months to 1 year after radiation completion), we calculated and stratified the time between the last radiation and time of perfusion scan collected to assess whether this could have affected the prediction of tumor percentage. The incorporation of PWI into the standard follow-up protocol for high-grade patients remains a challenge [11,12,13,14,15]. Perhaps with standardized and validated grading criteria such as ours, the integration of a follow-up PWI protocol may be considered. 

Many studies have retrospectivelyand prospectively confirmed the efficacy of PWI in diagnosing tumor recurrence [10]. In particular, they have used Cohen’s kappa (κ) specifically as a reliable metric to compare raters. Our κ value (0.272) was discrepant from these studies, however. This difference can be attributed to the studies using similar raters within a group to negate intra-rater variation and not assess inter-group differences. For example, studies have compared neuroradiologists and multi-disciplinary treatment teams or just between neuro-oncology specialized neuroradiologists but never between raters with a novel grading rubric and those without [10,16].

This study is the first to address such a discrepancy, thus yielding a lower κ value. The PWI analysis performed by the proposed grading criteria could accurately predict tumor recurrence versus treatment effects, similar to previous studies on PWI. Although PWI is a powerful tool for CNS lesion diagnosis, standardized grading criteria must be present in order for PWI to be useful. A limitation is that the grading rubric was used by only a single reader, who likely read these cases with more granularity and care, which may have contributed to the improvement using this method. However, the structured rating scale is easily teachable and can be extended to other readers in the future. We hope to bridge the gap for this disconnect with our grading scale and further generalize our findings through subsequent studies with greater numbers of raters and external validation from other sites.

## Figures and Tables

**Table 1:** Criteria used for assigning perfusion scores based on rCBV with corresponding examples.

Perfusion Scoring	Structured Scoring Criteria
Not interpretable	No useful perfusion information available Perfusion information obscured by susceptibility artifacts from blood products Technical failure of perfusion (e.g., failed bolus timing)
High perfusion	rCBV similar to the cerebral cortex in the majority (>50%) of the area with concerning abnormal FLAIR and post-contrast enhancement
Intermediate perfusion	rCBV between the cerebral cortex and normal white matter in the majority (>50%) of the area with concerning abnormal FLAIR and post-contrast enhancement OR rCBV similar to the cerebral cortex in portions of the area (<50%) of concerning abnormal FLAIR and post-contrast enhancement
Low perfusion	rCBV similar to or less than normal white matter in the majority (>50%) of the area with concerning abnormal FLAIR and post-contrast enhancement and no areas of rCBV similar to the cerebral cortex

**Table 2:** Pre-operative, operative, and post-operative details.

Variable	Level	N (%) = 55
Pre-operative		
Age	Mean (SD)	53.5 (12.2)
Gender	Male	32 (58.2)
Pre-operative BMI	Mean (SD)	28.7 (4.9)
Pre-operative KPS	Mean (SD)	80.3 (8.0)
Pre-operative MRS	Mean (SD)	1.4 (0.5)
Symptoms at Surgery	Asymptomatic/Surgery Based on Imaging Progression	25 (45.5)
	Some Symptoms	17 (30.9)
	Major Symptoms/Seizures	13 (23.6)
Edema		23 (41.2)
Mass Effect		29 (52.7)
Immunotherapy Before Surgery		10 (18.2)
Clinical Trial Before Surgery		5 (9.1)
Chemotherapy Before Surgery		49 (89.1)
Steroid Trial Before Surgery		43 (78.2)
Frequency of Steroid Trials	Median (IQR)	2 (1–4)
Steroid Trial Dose closest to Surgery	Median (IQR)	7.5 (4–12)
Bevacizumab Before Surgery		5 (9.1)
Operative		
Operation	Craniotomy	50 (90.9)
	Biopsy	5 (9.1)
Extent of Resection (N = 50)	Gross-Total Resection	27 (54.0)
	Sub-Total Resection	23 (46.0)
Lobe of Brain	Frontal	23 (41.8)
	Temporal	14 (25.5)
	Parietal	13 (23.6)
	Occipital	5 (9.1)
Percentage—Therapy-related Effects	Median (IQR)	50 (25–90)
Percentage—Residual/Recurrent Tumor	Median (IQR)	50 (10–75)
Post-operative		
Radiation Post Surgery		10 (18.2)
Chemotherapy Post Surgery		28 (50.9)
Immunotherapy Post Surgery		3 (5.5)
Clinical Trial Post Surgery		4 (7.3)
Post-operative KPS	Mean (SD)	73.5 (5.4)
Surgical Complications		7 (12.7)
Discharge KPS	Mean (SD)	82.9 (7.6)
Discharge MRS	Mean (SD)	1.25 (0.5)

**Table 3:** Association between the Perfusion checked for the control group in predicting the percentage of tumor found in the biopsied brain tissue.

Total N = 54	Tumor ≤ 25% N (%) = 18	Tumor 26–75% N (%) = 23	Tumor > 75% N (%) = 13	*p*-Value
Perfusion Read				0.734
High	11 (61.1)	18 (78.3)	9 (69.2)	
Intermediate	5 (27.8)	3 (13.0)	2 (15.4)	
Low	2 (11.1)	2 (8.7)	2 (15.4)	
Stratified by the Previous History of Metastasis				
**Total N = 12**	**Tumor ≤ 25% N (%) = 7**	**Tumor 26–75% N (%) = 2**	**Tumor > 75% N (%) = 3**	***p*-Value**
Perfusion Read				0.849
High	4 (57.1)	1 (50.0)	1 (33.3)	
Intermediate	2 (28.6)	1 (50.0)	2 (66.7)	
Low	1 (14.3)	0	0	
Stratified by the Previous History of Glioma				
**Total N = 41**	**Tumor ≤ 25% N(%) = 10**	**Tumor 26–75% N(%) = 21**	**Tumor > 75% N(%) = 10**	***p*-Value**
Perfusion Read				0.661
High	7 (70.0)	17 (81.0)	8 (80.0)	
Intermediate	2 (20.0)	2 (9.52)	0	
Low	1 (10.0)	2 (9.52)	2 (20.0)	

**Table 4:** Association between the perfusion checked for the experimental group in predicting the percentage of tumor found in the biopsied brain tissue.

Total N = 55	Tumor ≤ 25% N (%) = 18	Tumor 26–75% N (%) = 24	Tumor > 75% N (%) = 13	*p*-Value
Perfusion Read				0.014
High	3 (16.7)	15 (62.5)	8 (61.5)	
Intermediate	9 (50.0)	7 (29.2)	2 (15.4)	
Low	6 (33.3)	2 (8.3)	3 (23.1)	
Stratified by the Previous History of Metastasis				
**Total N = 12**	**Tumor ≤ 25% N (%) = 7**	**Tumor 26–75% N (%) = 2**	**Tumor > 75% N(%) = 3**	***p*-Value**
Perfusion Read				0.636
High	2 (28.6)	0	2 (66.7)	
Intermediate	3 (42.9)	2 (100.0)	1 (33.3)	
Low	2 (28.6)	0	0	
Stratified by the Previous History of Glioma				
**Total N = 42**	**Tumor ≤ 25% N (%) = 10**	**Tumor 26–75% N (%) = 22**	**Tumor > 75% N (%) = 10**	***p*-Value**
Perfusion Read				0.023
High	1 (10.0)	15 (68.2)	6 (60.0)	
Intermediate	5 (50.0)	5 (22.7)	1 (10.0)	
Low	4 (40.0)	2 (9.09)	3 (30.0)	

## Data Availability

Due to privacy and ethical restrictions, the patient data supporting the reported results in this study cannot be shared publicly. Access to the anonymized data may be granted upon reasonable request and subject to appropriate ethical approval.
